# Supplementary material for: Thalamus sends information about arousal but not valence to the amygdala
Source: Psychopharmacology (Berl). 2022 Dec 16;240(3):477–99. doi: 10.1007/s00213-022-06284-5 (PMC9928937; doi:10.1007/s00213-022-06284-5)
Supplement: Supplementary file 1 — (DOCX 37 KB) [file 213_2022_6284_MOESM1_ESM.docx]

**Supplemental Fig. 1 Viable Electrode Placements in MGN and BLA for electrophysiology recordings**

(a, b) Electrolytic lesion sites indicate recording location in MGN (top) and BLA (bottom). Experimental subjects with optrode placements outside of MGN were excluded from analysis. Subjects with correct electrode placements in MGN did not all have viable electrode placements in BLA. Electrode placements outside BLA are not shown, as data from these channels was excluded from analysis.

**Supplemental Fig. 2 MGN and BLA Neurons Exhibit Similar Rate Change Trial During Discrimination Learning**

(a, b) Representative raster and PSTH (50 ms bins) for the period -2 s before cue to +4 s after cue for (a) MGN and (b) BLA neuron; baseline early trials (shaded red) and conditioning (shaded green) periods, tone onset shown by red line. Dotted box in PSTH represents the 150 ms interval over which state space analysis was computed. Lower plots show probabilistic estimate of the trial at which neuron undergoes a rate change using state-space analysis; black arrow indicates rate change trial number.

(c) Representative example of randomly selected trial matched pairs of rate change trial number for MGN and BLA neurons for CS-A. The distributions do not exhibit separation in the rate change trial (Kolmogorov–Smirnov test inconclusive for 25 randomly selected matched trials); thus, our findings are inconclusive as to whether one neuronal population learns before the other.

(d) Cue-responsive MGN subpopulations; 46% of cells respond to task. Of these,1% (n=1) changed with conditioning.

(e) Cue-responsive BLA subpopulations; 25% responded to task. Of these 12% (n=2) changed with conditioning.

(f) Spike raster and PSTH for single MGN cell that changed with conditioning. This cell was initially excited to cue and weakened with conditioning.

(g) Spike raster and PSTH for both BLA cells that changed with conditioning. Both cells were initially excited to cue and weakened with conditioning.

Supplemental Methods

*MGN and BLA Neurons Exhibit Similar Encoding Rates During Discrimination Learning and Few Neurons Exhibit Conditioning during Punishment Learning.*

We applied a state-space analysis to all neurons in each brain region in order to characterize the neural dynamics in the MGN and the BLA (Supplemental Figure 2a, b). Such models allow accurate estimation of instantaneous firing rate as well as the ability to identify the trial at which the firing rate changes within a statistical framework; the latter “rate-change trial” represents the point at which a neuron encodes the learned association. We hypothesized that the MGN would encode this information before the BLA but found no significant difference between their rate change trial distributions (Kolmogorov–Smirnov test, matched pairs, n.s., Supplemental Figure 2d). In other words, the MGN does not appear to learn an association before passing on information downstream.

To characterize whether the neural response changed over the course of the discrimination session, early trials and late trials of discrimination session data were compared (Supplemental Figure 2d). Due to limitations in the experimental paradigm, a habituation phase was not expressly included in the trial structure. To interrogate whether neurons changed with conditioning, we denote trials 1-10 of the punishment cue to act as the habituation phase during discrimination learning, while trials 11-35 form the conditioning phase. There were proportionally more cue-responsive neurons in the MGN than in the BLA (MGN: 46%, 82/179, N = 12; BLA: 25%, 16/63, N = 6). Of these cue-responsive neurons, proportionally fewer MGN neurons experienced a change with conditioning than BLA neurons (MGN: 1%, 1/82, N = 12; BLA: 12%, 2/16, N=6). The sole MGN neuron that underwent a change in conditioning was excited to the cue and the mean z-score response weakened with conditioning (Supplemental Figure 2f). The two BLA neurons that changed with conditioning were both excited to the cue and weakened the magnitude response with conditioning (Supplemental Figure 2g).

*Calculation of firing rate and rate change trial*

Firing rate was modeled using the state-space approach described in Smith et al. (2010) and was modified as described in (Allsop et al., 2018) to find the rate change trial. The MATLAB code for computing state-space analysis of neural firing may be downloaded from <http://annecsmith.net/firingrates.html>. To determine whether a cell changed with conditioning over the course of the task, the first 10 trials of punishment data were assigned as the habituation phase and the last 10 trials as the conditioning phase. An ANOVA across four time-windows (two baseline and two response) was computed to see if there was a difference between the average firing rate in each window. We then compared the two experimental windows in post-hoc comparison tests. If there was a statistically significant difference between the firing rate across both experimental response windows, that unit was identified as changing with conditioning (Task ∆).
