# Supplementary material for: Thalamus sends information about arousal but not valence to the amygdala
Source: Psychopharmacology (Berl). 2022 Dec 16;240(3):477–99. doi: 10.1007/s00213-022-06284-5 (PMC9928937; doi:10.1007/s00213-022-06284-5)

Supplementary Figure 1

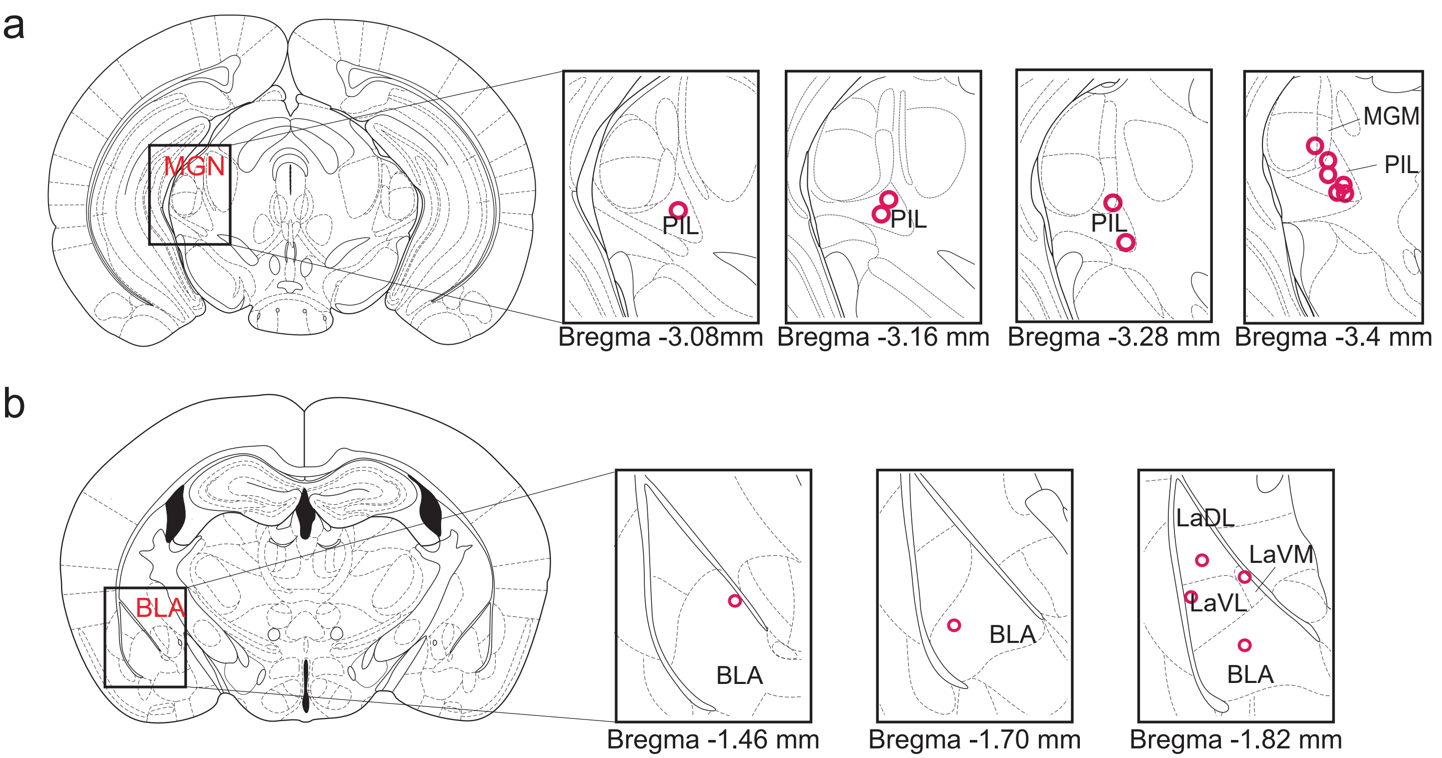

MGM	medial division of the medial geniculate nucleus
PIL	posterior interlaminar nucleus
BLA	basolateral amygdala
LaDL	lateral amygdala medial subdivision
LaVM	lateral amygdala, ventromedial subdivision
LaVL	lateral amygdala ventrolateral subdivision

Supplementary Figure 2

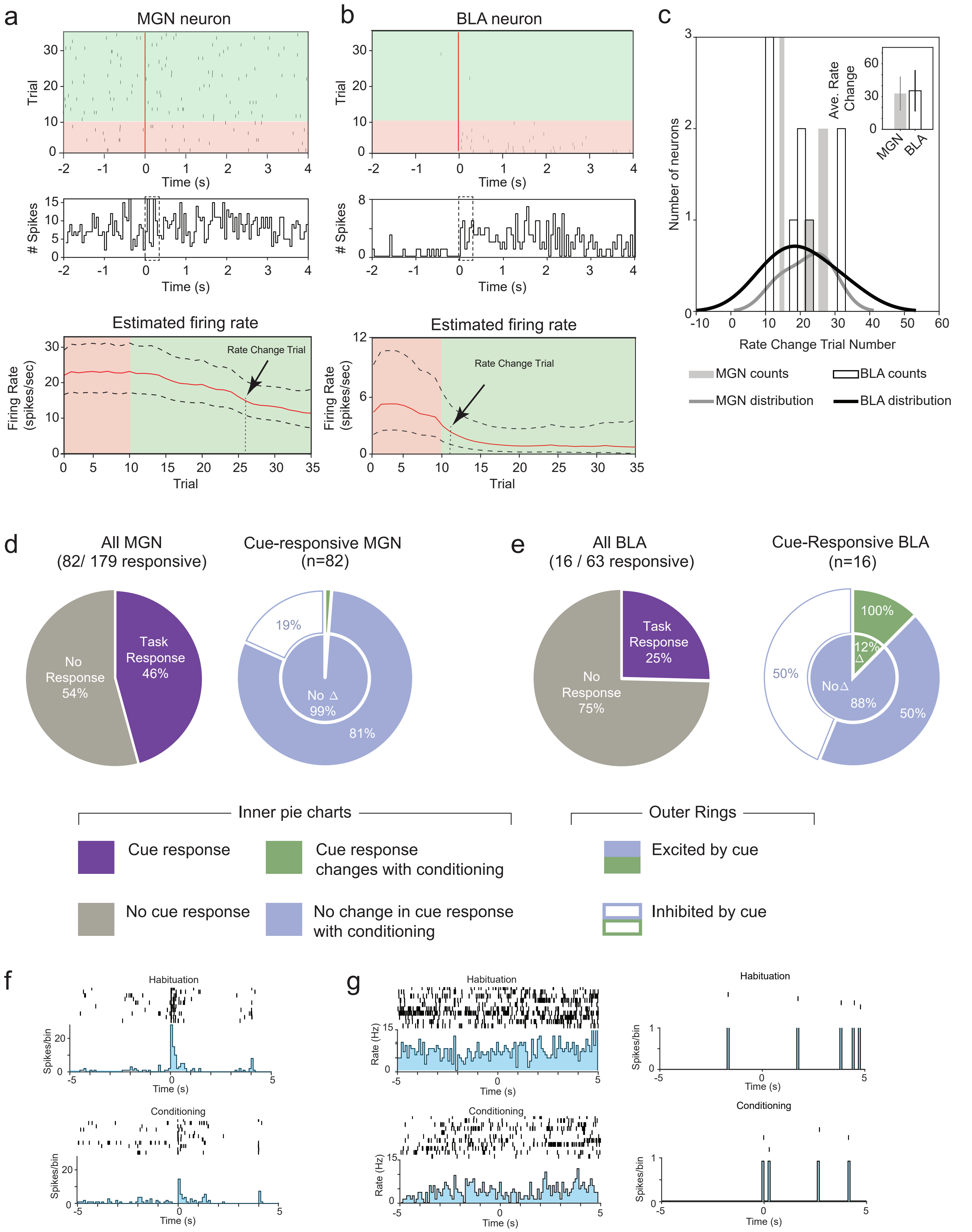

Supplement: Supplementary file 2 — (PDF 300 KB) [file 213_2022_6284_MOESM2_ESM.pdf]
